# Crystal structure of 2-meth­oxy-1-nitro­naphthalene

**DOI:** 10.1107/S2056989015016114

**Published:** 2015-09-12

**Authors:** Hasna Yassine, Mostafa Khouili, Lahcen El Ammari, Mohamed Saadi, El Mostafa Ketatni

**Affiliations:** a Laboratoire de Chimie Organique et Analytique, Université Sultan Moulay Slimane, Faculté des Sciences et Techniques, BP 523, 23000 Béni-Mellal, Morocco; b Laboratoire de Chimie du Solide Appliquée, Faculté des Sciences, Université Mohammed V, Avenue Ibn Battouta, BP 1014, Rabat, Morocco; c Laboratoire de Spectrochimie Applique et Environnement, Université Sultan Moulay Slimane, Faculté des Sciences et Techniques, BP 523, 23000 Béni-Mellal, Morocco

**Keywords:** crystal structure, naphthalene derivative, weak C—H⋯O inter­actions, π–π stacking

## Abstract

The asymmetric unit of the title compound, C_11_H_9_NO_3_, contains two mol­ecules, *A* and *B*. In mol­ecule *A*, the dihedral angle between the planes of the naphthalene ring system (r.m.s. deviation = 0.003 Å) and the nitro group is 89.9 (2)°, and the C atom of the meth­oxy group deviates from the naphthyl plane by 0.022 (2) Å. Equivalent data for mol­ecule *B* are 0.008 Å, 65.9 (2)° and −0.198 (2) Å, respectively. In the crystal, mol­ecules are linked by weak C—H⋯O inter­actions, forming [100] chains of alternating *A* and *B* mol­ecules. Weak aromatic π–π stacking contacts, with a range of centroid–centroid distances from 3.5863 (9) to 3.8048 (9) Å, are also observed.

## Related literature   

For biological activities of naphthalene derivatives, see: Wright *et al.* (2000 ▸); Rokade & Sayyed (2009 ▸); Upadhayaya *et al.* (2010 ▸). For the title compound as an inter­mediate in the synthesis of anti­pyretic drugs, see: Stoylkova *et al.* (2000 ▸); Govindarajana *et al.* (2011 ▸); Kirumakki *et al.* (2004 ▸); Yadav *et al.* (1998 ▸). For a related structure, see: Wannalerse *et al.* (2013 ▸).
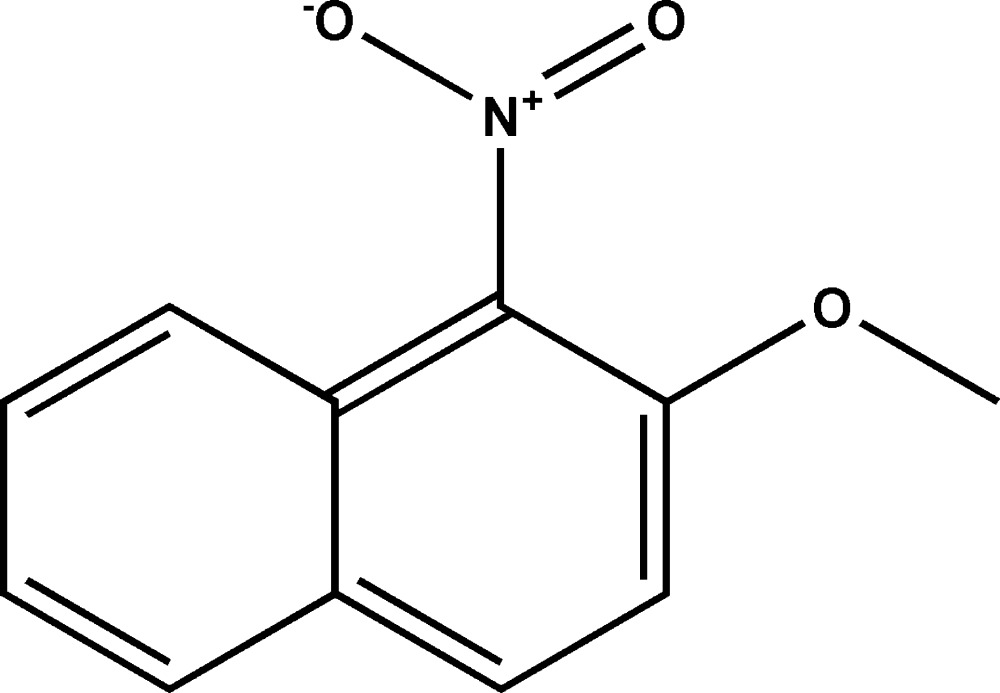


## Experimental   

### Crystal data  

C_11_H_9_NO_3_
*M* *_r_* = 203.19
Triclinic, 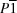

*a* = 9.1291 (4) Å
*b* = 10.2456 (4) Å
*c* = 10.5215 (4) Å
α = 86.390 (2)°
β = 82.964 (2)°
γ = 85.801 (2)°
*V* = 972.63 (7) Å^3^
*Z* = 4
Mo *K*α radiation
μ = 0.10 mm^−1^
*T* = 296 K
0.39 × 0.32 × 0.24 mm

### Data collection  

Bruker X8 APEXII CCD diffractometer
Absorption correction: multi-scan (*SADABS*; Bruker, 2009 ▸) *T*_min_ = 0.676, *T*_max_ = 0.746
34901 measured reflections
5450 independent reflections
3446 reflections with *I* > 2σ(*I*)
*R*_int_ = 0.038

### Refinement  

*R*[*F* ^2^ > 2σ(*F* ^2^)] = 0.048
*wR*(*F* ^2^) = 0.141
*S* = 1.04
5450 reflections
272 parameters
H-atom parameters constrained
Δρ_max_ = 0.20 e Å^−3^
Δρ_min_ = −0.16 e Å^−3^

### 

Data collection: *APEX2* (Bruker, 2009 ▸); cell refinement: *SAINT* (Bruker, 2009 ▸); data reduction: *SAINT*; program(s) used to solve structure: *SHELXS97* (Sheldrick, 2008 ▸); program(s) used to refine structure: *SHELXL97* (Sheldrick, 2008 ▸); molecular graphics: *ORTEP-3 for Windows* (Farrugia, 2012 ▸); software used to prepare material for publication: *PLATON* (Spek, 2009 ▸) and *publCIF* (Westrip, 2010 ▸).

## Supplementary Material

Crystal structure: contains datablock(s) I. DOI: 10.1107/S2056989015016114/hb7477sup1.cif

Structure factors: contains datablock(s) I. DOI: 10.1107/S2056989015016114/hb7477Isup2.hkl

Supporting information file. DOI: 10.1107/S2056989015016114/hb7477Isup3.cml

**Fig. 1.**
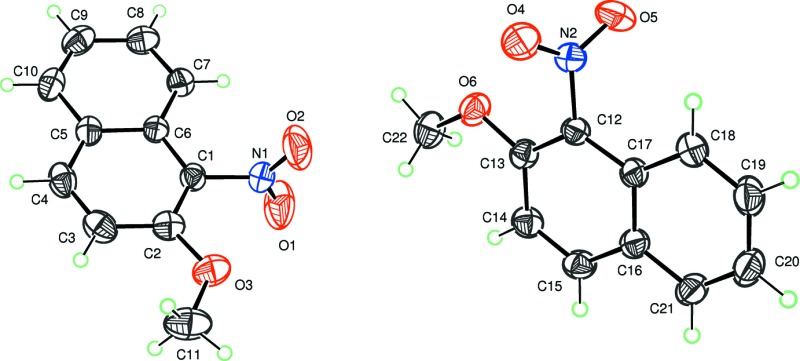
: A view of the molecule of the title compound, showing displacement ellipsoids drawn at the 50% probability level. H atoms are represented as small circles.

. DOI: 10.1107/S2056989015016114/hb7477fig1.tif
A view of the mol­ecule of the title compound, showing displacement ellipsoids drawn at the 50% probability level. H atoms are represented as small circles.

**Fig. 2.**
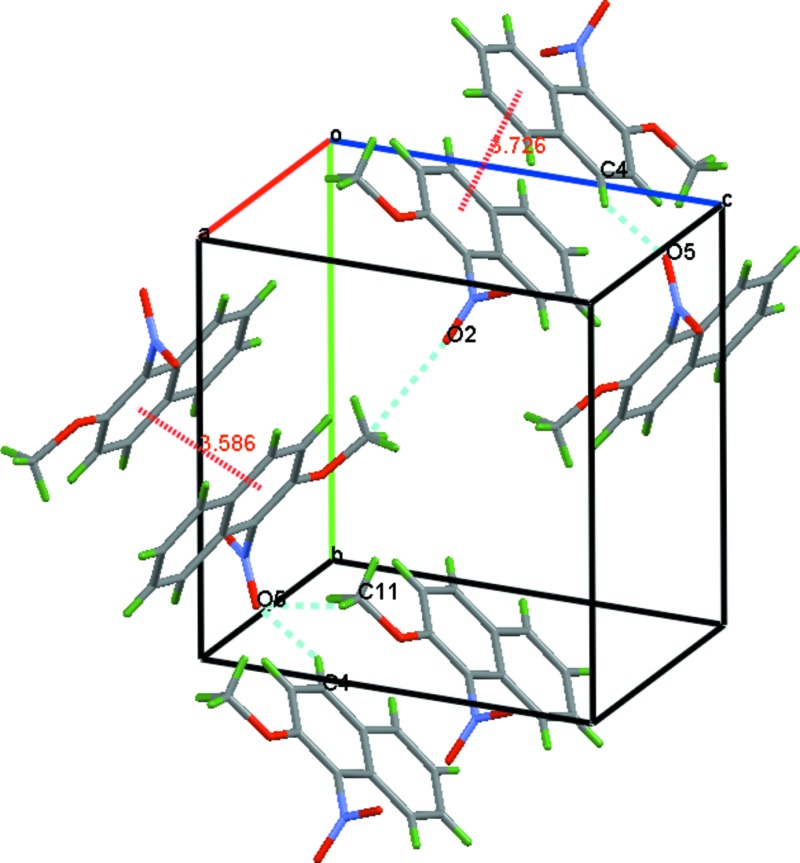
: Partial crystal packing for the title compound showing molecules linked by hydrogen bonds as blue dashed lines and π–π contacts between the naphthalene rings (red dashed lines).

. DOI: 10.1107/S2056989015016114/hb7477fig2.tif
Partial crystal packing for the title compound showing mol­ecules linked by hydrogen bonds as blue dashed lines and π–π contacts between the naphthalene rings (red dashed lines).

CCDC reference: 1421062

Additional supporting information:  crystallographic information; 3D view; checkCIF report

## Figures and Tables

**Table 1:** Hydrogen-bond geometry (, )

*D*H*A*	*D*H	H*A*	*D* *A*	*D*H*A*
C4H4O5^i^	0.93	2.57	3.409(2)	150
C11H11*A*O5^ii^	0.96	2.60	3.462(3)	150

Symmetry codes: (i) 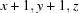; (ii) 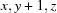.

## supplementary crystallographic information

### S1. Comment

Naphthalene derivatives have been extensively employed in many fields, for example, as a colorant, explosive, disinfectant, insecticide, auxin plant hormone and play a role in the chemical defence against biological (Wright *et al.*, 2000) and have diverse and interesting antibiotic properties (Rokade & Sayyed, 2009; Upadhayaya *et al.* 2010). 2-Methoxynaphthalene is an important intermediate used in the production of naproxen. It is widely used a non-steroidal anti-inflammatory, analgesic and antipyretic drug (Stoylkova *et al.*, 2000, Govindarajana *et al.*, 2011, Kirumakki *et al.*, 2004; Yadav *et al.*, 1998). Nitration of 1-methoxynaphthalene with bismuth nitrate in CH_2_Cl_2_ gives a compound (I) and describes its structure here.

The asymmetric unit of the title compound consists of two crystallographically independent molecules of nearly similar geometry as shown in Fig. 1. Bond lengths and angles of the title compound are comparable with that found in the similar structure (Wannalerse *et al.*, 2013). In the first (O1O2O3N1C1–C11) and second (O4O5O6N2C12–C22) molecules, the dihedral angles between the nitro group and the attached naphthalene system are 89.9 (2)° and 65.9 (2)°, respectively. The two naphthalene rings belonging to the both molecules form a dihedral angle of 72.02 (5)°.

In the crystal, the molecules are linked together by weak C—H···O interactions. Moreover, the π–π contacts between the naphthalene rings, may further consolidate the structure, with range of centroid– centroid distances = 3.5863 (9)—3.8048 (9) Å.

### S2. Experimental

2-Methoxynaphtalene (500 mg, 3.164 mmol) and silica gel (500 mg) was added to a suspension of bismuth nitrate pentahydrate (1.2 eqv.) in CH_2_Cl_2_ (20 ml). The mixture was refluxed for 6 h. After cooling to room temperature, the reaction mixture was filtered and watched with CH_2_Cl_2_, the filtrate obtained was concentrated, and the resulting residue was purified by column chromatography using EtOAc-Hexane (1:9 *v*/*v*). The title compound was recrystallized from the solvent mixture ethyl acetate/hexane to yield orange block crystals (yield: 74%).

### S3. Refinement

All H atoms could be located in a difference Fourier map. However, they were placed in calculated positions with C—H = 0.93–0.96 Å, and refined as riding on their parent atoms with *U*_iso_(H) = 1.2 *U*_eq_ for aromatic and *U*_iso_(H) = 1.5 *U*_eq_ (C) for methyl. Two outlier reflections, (-7 3 0) and (-1 - 3 2), were omitted in the last cycles of refinement.

### Crystal data

**Table:**

C_11_H_9_NO_3_	*Z* = 4
*M**_r_* = 203.19	*F*(000) = 424
Triclinic, *P*1	*D*_x_ = 1.388 Mg m^−^^3^
*a* = 9.1291 (4) Å	Mo *K*α radiation, λ = 0.71073 Å
*b* = 10.2456 (4) Å	Cell parameters from 3506 reflections
*c* = 10.5215 (4) Å	θ = 1.7–30.0°
α = 86.390 (2)°	µ = 0.10 mm^−^^1^
β = 82.964 (2)°	*T* = 296 K
γ = 85.801 (2)°	Block, orange
*V* = 972.63 (7) Å^3^	0.39 × 0.32 × 0.24 mm

### Data collection

**Table:**

Bruker X8 APEXII CCD diffractometer	5450 independent reflections
Radiation source: fine-focus sealed tube	3446 reflections with *I* > 2σ(*I*)
Graphite monochromator	*R*_int_ = 0.038
φ and ω scans	θ_max_ = 29.6°, θ_min_ = 2.0°
Absorption correction: multi-scan (*SADABS*; Bruker, 2009)	*h* = −12→12
*T*_min_ = 0.676, *T*_max_ = 0.746	*k* = −14→14
34901 measured reflections	*l* = −14→14

### Refinement

**Table:**

Refinement on *F*^2^	Hydrogen site location: inferred from neighbouring sites
Least-squares matrix: full	H-atom parameters constrained
*R*[*F*^2^ > 2σ(*F*^2^)] = 0.048	*w* = 1/[σ^2^(*F*_o_^2^) + (0.0511*P*)^2^ + 0.2462*P*] where *P* = (*F*_o_^2^ + 2*F*_c_^2^)/3
*wR*(*F*^2^) = 0.141	(Δ/σ)_max_ < 0.001
*S* = 1.04	Δρ_max_ = 0.20 e Å^−^^3^
5450 reflections	Δρ_min_ = −0.15 e Å^−^^3^
272 parameters	Extinction correction: *SHELXL97* (Sheldrick, 2008), Fc^*^=kFc[1+0.001xFc^2^λ^3^/sin(2θ)]^-1/4^
0 restraints	Extinction coefficient: 0.011 (2)

### Special details

**Table:**

Geometry. All e.s.d.'s (except the e.s.d. in the dihedral angle between two l.s. planes) are estimated using the full covariance matrix. The cell e.s.d.'s are taken into account individually in the estimation of e.s.d.'s in distances, angles and torsion angles; correlations between e.s.d.'s in cell parameters are only used when they are defined by crystal symmetry. An approximate (isotropic) treatment of cell e.s.d.'s is used for estimating e.s.d.'s involving l.s. planes.

### Fractional atomic coordinates and isotropic or equivalent isotropic displacement parameters (Å^2^)

**Table:**

	*x*	*y*	*z*	*U*_iso_*/*U*_eq_	
C1	0.82138 (17)	0.83303 (15)	0.59386 (15)	0.0445 (3)	
C2	0.76837 (19)	0.92960 (16)	0.67524 (16)	0.0512 (4)	
C3	0.8714 (2)	0.99956 (17)	0.72711 (17)	0.0582 (4)	
H3	0.8389	1.0650	0.7832	0.070*	
C4	1.0189 (2)	0.97177 (17)	0.69542 (16)	0.0562 (4)	
H4	1.0854	1.0201	0.7298	0.067*	
C5	1.07461 (18)	0.87266 (15)	0.61254 (15)	0.0464 (4)	
C6	0.97310 (17)	0.79982 (14)	0.55904 (14)	0.0416 (3)	
C7	1.02802 (19)	0.70018 (16)	0.47545 (16)	0.0515 (4)	
H7	0.9625	0.6519	0.4398	0.062*	
C8	1.1760 (2)	0.67452 (19)	0.44694 (18)	0.0605 (5)	
H8	1.2107	0.6085	0.3920	0.073*	
C9	1.2771 (2)	0.7456 (2)	0.49869 (19)	0.0633 (5)	
H9	1.3782	0.7266	0.4781	0.076*	
C10	1.2280 (2)	0.84240 (19)	0.57914 (18)	0.0580 (5)	
H10	1.2961	0.8895	0.6128	0.070*	
C11	0.5602 (3)	1.0473 (2)	0.7849 (2)	0.0859 (7)	
H11A	0.4542	1.0518	0.7914	0.129*	
H11B	0.5951	1.1311	0.7543	0.129*	
H11C	0.5912	1.0242	0.8678	0.129*	
C12	0.12086 (16)	0.31177 (13)	0.90308 (14)	0.0391 (3)	
C13	0.19472 (16)	0.40531 (14)	0.82703 (14)	0.0411 (3)	
C14	0.11092 (19)	0.51331 (15)	0.77661 (15)	0.0477 (4)	
H14	0.1581	0.5779	0.7242	0.057*	
C15	−0.03834 (18)	0.52299 (15)	0.80456 (15)	0.0483 (4)	
H15	−0.0915	0.5959	0.7721	0.058*	
C16	−0.11572 (17)	0.42663 (14)	0.88090 (14)	0.0424 (3)	
C17	−0.03443 (16)	0.31586 (14)	0.93202 (14)	0.0391 (3)	
C18	−0.11305 (19)	0.22006 (16)	1.00938 (17)	0.0515 (4)	
H18	−0.0618	0.1475	1.0446	0.062*	
C19	−0.2632 (2)	0.23339 (18)	1.0326 (2)	0.0612 (5)	
H19	−0.3135	0.1690	1.0827	0.073*	
C20	−0.34344 (19)	0.34223 (19)	0.9822 (2)	0.0618 (5)	
H20	−0.4460	0.3497	0.9990	0.074*	
C21	−0.27129 (18)	0.43676 (17)	0.90895 (18)	0.0532 (4)	
H21	−0.3251	0.5093	0.8767	0.064*	
C22	0.4201 (2)	0.47481 (19)	0.71352 (18)	0.0615 (5)	
H22A	0.5244	0.4516	0.7073	0.092*	
H22B	0.4010	0.5629	0.7403	0.092*	
H22C	0.3862	0.4688	0.6314	0.092*	
N1	0.20980 (15)	0.20198 (13)	0.95773 (14)	0.0483 (3)	
N2	0.71338 (16)	0.76148 (15)	0.53852 (16)	0.0571 (4)	
O1	0.6734 (2)	0.8025 (2)	0.43824 (19)	0.1118 (7)	
O2	0.6708 (2)	0.66298 (17)	0.5941 (2)	0.1043 (6)	
O3	0.61970 (14)	0.95078 (14)	0.69777 (14)	0.0703 (4)	
O4	0.29054 (17)	0.22522 (13)	1.03577 (15)	0.0785 (4)	
O5	0.19774 (17)	0.09259 (12)	0.92409 (16)	0.0767 (4)	
O6	0.34371 (12)	0.38730 (11)	0.80514 (11)	0.0537 (3)	

### Atomic displacement parameters (Å^2^)

**Table:**

	*U*^11^	*U*^22^	*U*^33^	*U*^12^	*U*^13^	*U*^23^
C1	0.0446 (8)	0.0428 (8)	0.0464 (8)	−0.0077 (6)	−0.0072 (7)	0.0025 (6)
C2	0.0539 (10)	0.0513 (9)	0.0462 (9)	−0.0005 (7)	−0.0013 (7)	0.0027 (7)
C3	0.0772 (13)	0.0527 (9)	0.0447 (9)	−0.0034 (9)	−0.0046 (9)	−0.0084 (7)
C4	0.0708 (12)	0.0557 (10)	0.0465 (9)	−0.0177 (8)	−0.0179 (8)	−0.0006 (7)
C5	0.0510 (9)	0.0486 (8)	0.0410 (8)	−0.0110 (7)	−0.0117 (7)	0.0069 (7)
C6	0.0441 (8)	0.0415 (7)	0.0393 (8)	−0.0064 (6)	−0.0076 (6)	0.0052 (6)
C7	0.0527 (10)	0.0499 (9)	0.0526 (10)	−0.0041 (7)	−0.0081 (8)	−0.0046 (7)
C8	0.0582 (11)	0.0624 (11)	0.0580 (11)	0.0062 (8)	−0.0016 (8)	−0.0027 (8)
C9	0.0455 (10)	0.0768 (13)	0.0640 (12)	0.0008 (9)	−0.0028 (8)	0.0118 (10)
C10	0.0499 (10)	0.0688 (11)	0.0578 (11)	−0.0170 (8)	−0.0169 (8)	0.0122 (9)
C11	0.0839 (16)	0.0823 (15)	0.0800 (15)	0.0233 (12)	0.0198 (12)	−0.0064 (12)
C12	0.0404 (8)	0.0360 (7)	0.0417 (8)	−0.0007 (6)	−0.0080 (6)	−0.0039 (6)
C13	0.0390 (8)	0.0445 (8)	0.0403 (8)	−0.0033 (6)	−0.0047 (6)	−0.0046 (6)
C14	0.0524 (9)	0.0456 (8)	0.0442 (8)	−0.0048 (7)	−0.0055 (7)	0.0049 (6)
C15	0.0522 (9)	0.0444 (8)	0.0483 (9)	0.0032 (7)	−0.0121 (7)	0.0036 (7)
C16	0.0413 (8)	0.0445 (8)	0.0431 (8)	−0.0009 (6)	−0.0099 (6)	−0.0089 (6)
C17	0.0392 (8)	0.0386 (7)	0.0409 (8)	−0.0042 (6)	−0.0070 (6)	−0.0069 (6)
C18	0.0493 (9)	0.0441 (8)	0.0611 (10)	−0.0087 (7)	−0.0048 (8)	0.0001 (7)
C19	0.0505 (10)	0.0582 (10)	0.0746 (12)	−0.0188 (8)	0.0041 (9)	−0.0067 (9)
C20	0.0370 (9)	0.0710 (12)	0.0785 (13)	−0.0071 (8)	−0.0011 (8)	−0.0203 (10)
C21	0.0411 (9)	0.0570 (10)	0.0636 (11)	0.0021 (7)	−0.0125 (8)	−0.0125 (8)
C22	0.0487 (10)	0.0745 (12)	0.0592 (11)	−0.0146 (9)	0.0053 (8)	0.0020 (9)
N1	0.0435 (7)	0.0412 (7)	0.0595 (8)	−0.0011 (5)	−0.0071 (6)	0.0016 (6)
N2	0.0450 (8)	0.0572 (9)	0.0709 (10)	−0.0085 (6)	−0.0111 (7)	−0.0039 (7)
O1	0.1262 (16)	0.1253 (15)	0.1010 (13)	−0.0526 (12)	−0.0681 (12)	0.0190 (11)
O2	0.1055 (13)	0.0811 (11)	0.1351 (16)	−0.0523 (10)	−0.0365 (11)	0.0232 (10)
O3	0.0542 (8)	0.0768 (9)	0.0753 (9)	0.0099 (6)	0.0049 (6)	−0.0104 (7)
O4	0.0826 (10)	0.0663 (8)	0.0944 (11)	−0.0031 (7)	−0.0500 (9)	0.0107 (7)
O5	0.0839 (10)	0.0403 (7)	0.1082 (12)	0.0091 (6)	−0.0230 (8)	−0.0139 (7)
O6	0.0394 (6)	0.0609 (7)	0.0585 (7)	−0.0050 (5)	−0.0002 (5)	0.0050 (5)

### Geometric parameters (Å, º)

**Table:**

C1—C2	1.372 (2)	C12—N1	1.4678 (19)
C1—C6	1.411 (2)	C13—O6	1.3524 (18)
C1—N2	1.466 (2)	C13—C14	1.412 (2)
C2—O3	1.353 (2)	C14—C15	1.356 (2)
C2—C3	1.404 (3)	C14—H14	0.9300
C3—C4	1.360 (3)	C15—C16	1.408 (2)
C3—H3	0.9300	C15—H15	0.9300
C4—C5	1.406 (2)	C16—C21	1.412 (2)
C4—H4	0.9300	C16—C17	1.423 (2)
C5—C10	1.418 (2)	C17—C18	1.414 (2)
C5—C6	1.419 (2)	C18—C19	1.361 (2)
C6—C7	1.413 (2)	C18—H18	0.9300
C7—C8	1.358 (2)	C19—C20	1.400 (3)
C7—H7	0.9300	C19—H19	0.9300
C8—C9	1.396 (3)	C20—C21	1.357 (3)
C8—H8	0.9300	C20—H20	0.9300
C9—C10	1.360 (3)	C21—H21	0.9300
C9—H9	0.9300	C22—O6	1.427 (2)
C10—H10	0.9300	C22—H22A	0.9600
C11—O3	1.424 (2)	C22—H22B	0.9600
C11—H11A	0.9600	C22—H22C	0.9600
C11—H11B	0.9600	N1—O5	1.2129 (17)
C11—H11C	0.9600	N1—O4	1.2136 (18)
C12—C13	1.371 (2)	N2—O1	1.200 (2)
C12—C17	1.411 (2)	N2—O2	1.200 (2)
			
C2—C1—C6	124.01 (15)	O6—C13—C14	124.40 (14)
C2—C1—N2	117.77 (15)	C12—C13—C14	118.16 (14)
C6—C1—N2	118.21 (14)	C15—C14—C13	120.05 (14)
O3—C2—C1	116.87 (16)	C15—C14—H14	120.0
O3—C2—C3	125.16 (16)	C13—C14—H14	120.0
C1—C2—C3	117.96 (16)	C14—C15—C16	122.34 (14)
C4—C3—C2	120.12 (16)	C14—C15—H15	118.8
C4—C3—H3	119.9	C16—C15—H15	118.8
C2—C3—H3	119.9	C15—C16—C21	122.11 (14)
C3—C4—C5	122.44 (16)	C15—C16—C17	118.92 (14)
C3—C4—H4	118.8	C21—C16—C17	118.97 (14)
C5—C4—H4	118.8	C12—C17—C18	124.80 (14)
C4—C5—C10	123.00 (16)	C12—C17—C16	116.68 (13)
C4—C5—C6	118.74 (15)	C18—C17—C16	118.51 (14)
C10—C5—C6	118.27 (16)	C19—C18—C17	120.38 (16)
C1—C6—C7	124.13 (14)	C19—C18—H18	119.8
C1—C6—C5	116.72 (14)	C17—C18—H18	119.8
C7—C6—C5	119.15 (15)	C18—C19—C20	121.21 (17)
C8—C7—C6	120.33 (16)	C18—C19—H19	119.4
C8—C7—H7	119.8	C20—C19—H19	119.4
C6—C7—H7	119.8	C21—C20—C19	119.91 (16)
C7—C8—C9	121.14 (18)	C21—C20—H20	120.0
C7—C8—H8	119.4	C19—C20—H20	120.0
C9—C8—H8	119.4	C20—C21—C16	121.01 (16)
C10—C9—C8	120.03 (18)	C20—C21—H21	119.5
C10—C9—H9	120.0	C16—C21—H21	119.5
C8—C9—H9	120.0	O6—C22—H22A	109.5
C9—C10—C5	121.09 (17)	O6—C22—H22B	109.5
C9—C10—H10	119.5	H22A—C22—H22B	109.5
C5—C10—H10	119.5	O6—C22—H22C	109.5
O3—C11—H11A	109.5	H22A—C22—H22C	109.5
O3—C11—H11B	109.5	H22B—C22—H22C	109.5
H11A—C11—H11B	109.5	O5—N1—O4	123.26 (14)
O3—C11—H11C	109.5	O5—N1—C12	118.58 (14)
H11A—C11—H11C	109.5	O4—N1—C12	118.15 (13)
H11B—C11—H11C	109.5	O1—N2—O2	122.74 (17)
C13—C12—C17	123.81 (13)	O1—N2—C1	118.59 (15)
C13—C12—N1	117.52 (13)	O2—N2—C1	118.65 (16)
C17—C12—N1	118.66 (13)	C2—O3—C11	118.58 (17)
O6—C13—C12	117.43 (13)	C13—O6—C22	117.88 (13)

### Hydrogen-bond geometry (Å, º)

**Table:**

*D*—H···*A*	*D*—H	H···*A*	*D*···*A*	*D*—H···*A*
C4—H4···O5^i^	0.93	2.57	3.409 (2)	150
C11—H11*A*···O5^ii^	0.96	2.60	3.462 (3)	150

Symmetry codes: (i) *x*+1, *y*+1, *z*; (ii) *x*, *y*+1, *z*.
