# Supplementary figures and images for: Crystal structure of 2-meth­oxy-1-nitro­naphthalene
Source: Acta Crystallogr E Crystallogr Commun. 2015 Sep 12;71(Pt 10):o701–2. doi: 10.1107/S2056989015016114 (PMC4647346; doi:10.1107/S2056989015016114)

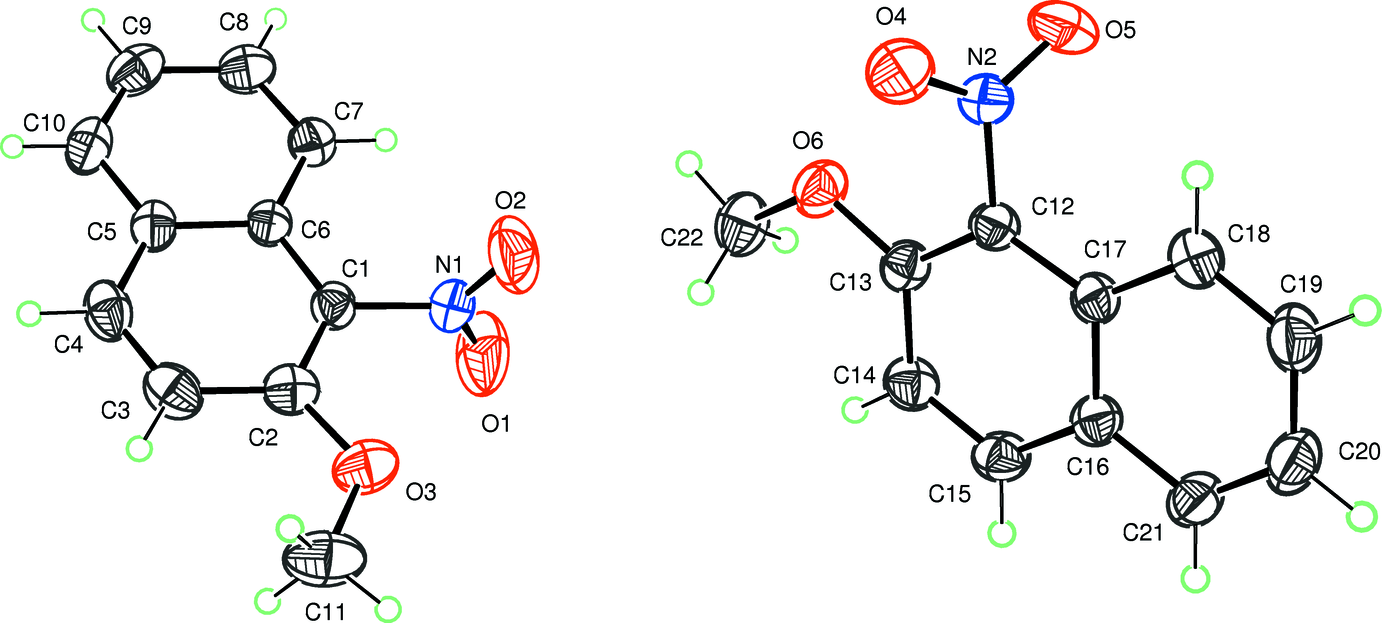

Supplement: Supplementary file 4 [file e-71-0o701-fig1.tif]

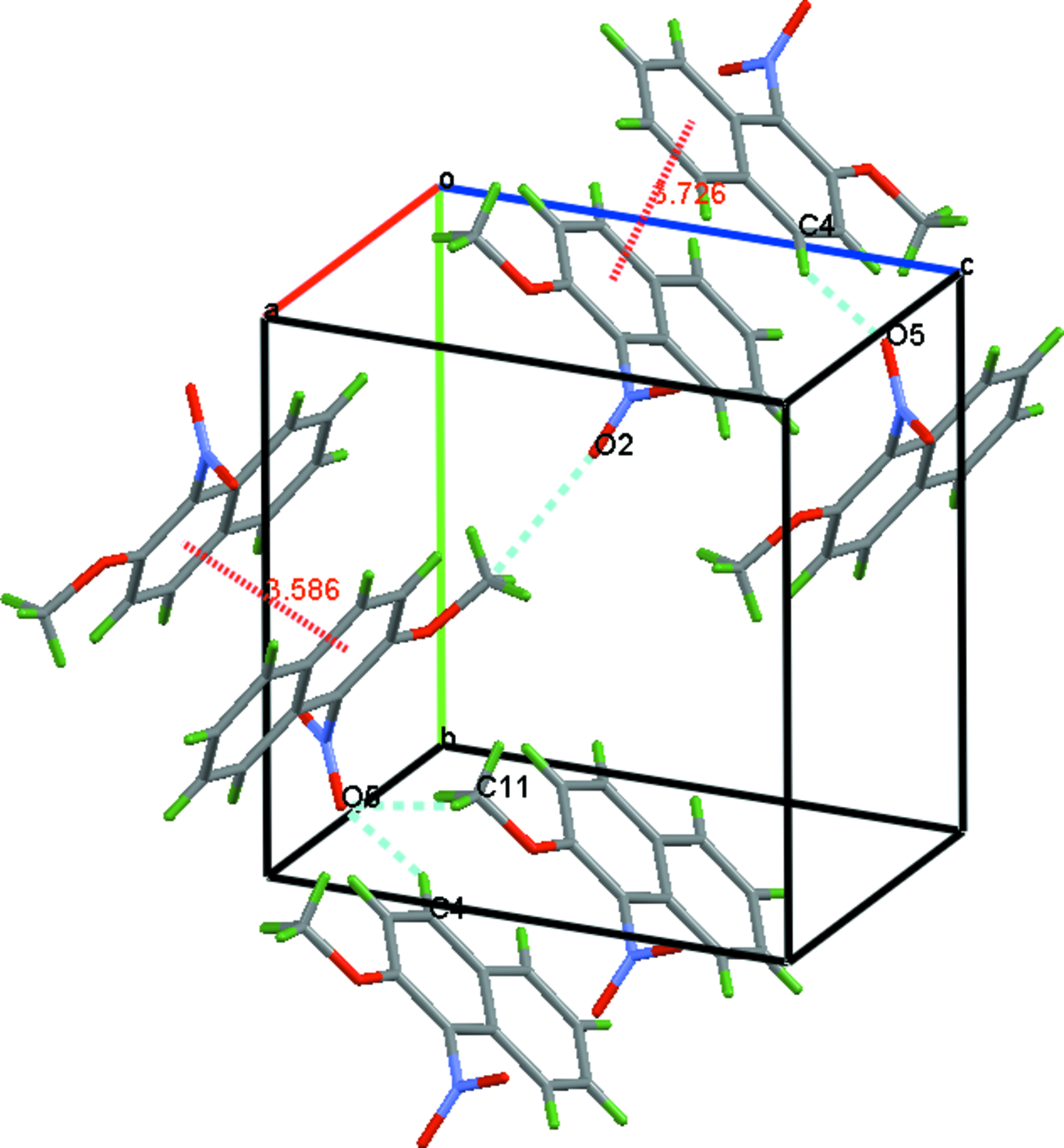

Supplement: Supplementary file 5 [file e-71-0o701-fig2.tif]
